# Association Between Levels of N-Terminal Pro-brain Natriuretic Peptide and Coronary Artery Lesion in Patients with Kawasaki Disease: A Systematic Review and Meta-analysis

**DOI:** 10.5152/ArchRheumatol.2025.11128

**Published:** 2025-06-23

**Authors:** Xinle Xu, Jin Wang

**Affiliations:** 1 Department of Pediatrics, Hangzhou Linping District Maternal and Child Health Hospital,Zhejiang, China; 2 Department of Child Health, Hangzhou Linping District Maternal and Child Health Hospital, Zhejiang, China

**Keywords:** Coronary artery lesions, Kawasaki disease, meta-analysis, NT-proBNP

## Abstract

**Background/Aims:**

Kawasaki disease (KD) is often complicated by coronary artery lesions (CAL). Identifying reliable biomarkers may improve early diagnosis and risk stratification for CAL, facilitating timely intervention. This study aims to investigate the diagnostic value of N-terminal pro-brain natriuretic peptide (NT-proBNP) in KD complicated with CAL.

**Materials and Methods:**

PubMed, Scopus, Web of Science, Embase, and the Cochrane Library databases were searched from inception to November 30, 2024 for English-language studies comparing NT-proBNP levels in KD patients with and without CAL. Diagnostic accuracy metrics for NT-proBNP in detecting CAL were also analyzed. The analysis was performed using a random-effects model. *I*² statistics assessed the heterogeneity. NT-proBNP levels reported as medians were converted to means using established formulas.

**Results:**

Nineteen studies involving 9017 participants showed significantly higher NT-proBNP levels in KD patients with CAL (pooled standardized mean differences = 1.889, 95% CI: 1.274 to 2.504, *P* < .001), with substantial heterogeneity (*I*² = 98.5%). Eighteen studies assessed diagnostic accuracy, yielding pooled sensitivity and specificity of 0.78 (95% CI: 0.68-0.85) and 0.78 (95% CI: 0.70-0.84), respectively. The diagnostic odds ratio was 12 (95% CI: 7-21), with an area under the receiver operating characteristic curve (AUROC) of 0.85 (95% CI: 0.81-0.88), indicating good diagnostic performance. However, heterogeneity remained significant (*I*² = 99%).

**Conclusion:**

N-terminal pro-brain natriuretic peptide is a promising biomarker for detecting CAL in KD, with good diagnostic accuracy. While elevated NT-proBNP levels correlate with CAL, its role is best realized as part of a multimodal diagnostic approach. Future research should focus on standardization and validation across diverse populations.

## Introduction

**Main Points**

- Kawasaki disease patients who develop coronary artery lesions (CAL) exhibit significantly higher NT-proBNP levels compared to those without CAL (pooled standardized mean difference = 1.889; 95% CI: 1.274–2.504; P < .001).
- The pooled diagnostic performance of NT-proBNP for detecting CAL shows good accuracy, with sensitivity = 0.78 (95% CI: 0.68–0.85), specificity = 0.78 (95% CI: 0.70–0.84), diagnostic odds ratio = 12 (95% CI: 7–21), and area under the ROC curve = 0.85 (95% CI: 0.81–0.88).
- There is substantial between-study heterogeneity in both NT-proBNP level comparisons (I² = 98.5%) and diagnostic accuracy metrics (I² = 99%), indicating variability in patient populations, assay methods, and CAL definitions that limits generalizability.
- Although NT-proBNP is a promising biomarker for identifying KD patients at risk of CAL, its standalone use is not advisable; it should be integrated into a multimodal diagnostic framework alongside clinical assessment and imaging, and standardized cutoff values need prospective validation.
- Evidence of publication bias (asymmetrical funnel plots and significant Egger’s/Deek’s tests) and reliance on observational studies underscore the need for large, prospective multicenter research to confirm these findings and refine NT-proBNP’s role in KD management.

Kawasaki disease (KD) is a self-limited vasculitis with unclear etiology that predominantly affects children under the age of 5^1^ and is accompanied by symptoms such as fever, rash, conjunctivitis, mucosal inflammation, cervical lymphadenopathy, etc.^2^ Despite the generally self-limiting nature of the disease, untreated cases can lead to devastating complications, most notably coronary artery lesions (CAL), including aneurysms and stenosis.^3^ While early diagnosis and appropriate treatment of KD significantly reduce the risk of coronary artery involvement, about 15%-25% of children with KD may still develop CAL even after treatment, underscoring the need for robust diagnostic and prognostic markers.^4^

N-terminal pro-brain natriuretic peptide (NT-proBNP), a cardiac biomarker widely used to assess cardiac stress and dysfunction, has garnered attention in recent years for its potential role in the context of KD.^5^ N-terminal pro-brain natriuretic peptide is a cleavage product of proBNP, secreted predominantly by cardiomyocytes in response to stretch.^6^ Increased levels of NT-proBNP have been consistently observed in children with KD, particularly in those with CAL, suggesting that this protein may be a marker for both disease severity and the risk of coronary complications.^7^ The non-specific nature of clinical manifestations in the early stages of KD often results in delayed diagnosis, especially in older children.^8^ N-terminal pro-brain natriuretic peptide, therefore, could serve as a critical adjunct in distinguishing KD from other febrile illnesses, particularly in incomplete or atypical cases.^9^

The link between NT-proBNP and CAL in KD is biologically plausible, given the systemic inflammatory milieu and potential myocardial involvement seen in the disease.^10^ Kawasaki disease is marked by an intense cytokine storm, endothelial dysfunction, and immune-mediated injury, which can impose significant stress on the cardiovascular system.^11^ Indeed, myocardial dysfunction, albeit often subclinical, is a recognized feature of acute KD and may contribute to the elevated NT-proBNP levels observed in affected children.^12^ Furthermore, NT-proBNP levels have been shown to correlate with inflammatory markers, further supporting its role as an integrative marker of inflammation and cardiac stress in KD.^13^

Despite the growing body of literature, the association between NT-proBNP levels and CAL in KD is still unclear due to the differences in patient populations, diagnostic criteria for KD, methodologies for NT-proBNP measurement, and definitions of CAL among studies. While some studies suggest that NT-proBNP levels are significantly higher in children who develop CAL, others have reported no such differences.^14,15^ Similarly, cutoff values for NT-proBNP that predict CAL vary widely across studies, limiting its clinical applicability as a reliable prognostic tool.^16,17^

Early identification of children at high risk for CAL is crucial for tailored anti-inflammatory therapies, closer monitoring, and long-term risk stratification, as children with CAL are at risk for ischemic heart disease later in life.^18^ Studying NT-proBNP in KD may also offer insights into the pathophysiological mechanisms underlying coronary involvement, paving the way for novel therapeutic targets.

Despite these promising data, existing syntheses have been limited by small sample sizes, variable assay methods, and inconsistent CAL definitions. Moreover, no prior analysis has simultaneously quantified the magnitude of NT-proBNP elevation in CAL, assessed its pooled diagnostic accuracy (sensitivity, specificity, area under the receiver operating characteristic curve [AUROC]), and systematically explored sources of between-study heterogeneity. Hence, this review was done to determine the diagnostic value of NT-proBNP in KD complicated with CAL.

## Materials and Methods

Preferred Reporting Items for Systematic Reviews and Meta-Analyses (PRISMA) 2020 guidelines were followed. The protocol was prospectively registered in the PROSPERO database (Registration Number: CRD42024620332). As this is a systematic review, ethic committee approval and informed consent was not needed.

## Eligibility Criteria

### Study Design

Studies (observational cohort, case-control, and cross-sectional) that evaluated NT-proBNP in KD patients were included. Case series, reports, review articles, editorials, and studies on non-humans were excluded.

### Participants

Studies enrolling children <18 years diagnosed with KD based on the American Heart Association or Japanese Circulation Society criteria were eligible. Studies focusing on KD patients with confirmed CAL or no coronary artery involvement were included.

### Exposure and Comparator

The primary exposure was elevated NT-proBNP levels. The comparator groups included KD patients without CAL.

### Outcomes

The primary outcomes were (1) Mean (SD) NT-proBNP levels in KD patients with and without CAL and (2) Diagnostic accuracy of NT-proBNP for detecting CAL.

### Search Strategy

PubMed, Scopus, Web of Science, Embase, and the Cochrane Library databases were screened from the inception of each database to November 30, 2024. Search terms included combinations of “Kawasaki Disease,” “NT-proBNP,” “coronary artery lesion,” and “diagnostic accuracy.” Boolean operators (“AND” and “OR”) were used to refine the search (see Supplementary Appendix). Additionally, bibliographies of relevant articles were manually searched for any missed studies. Only English-language articles were considered.

### Study Selection Process

**Figure 1.**
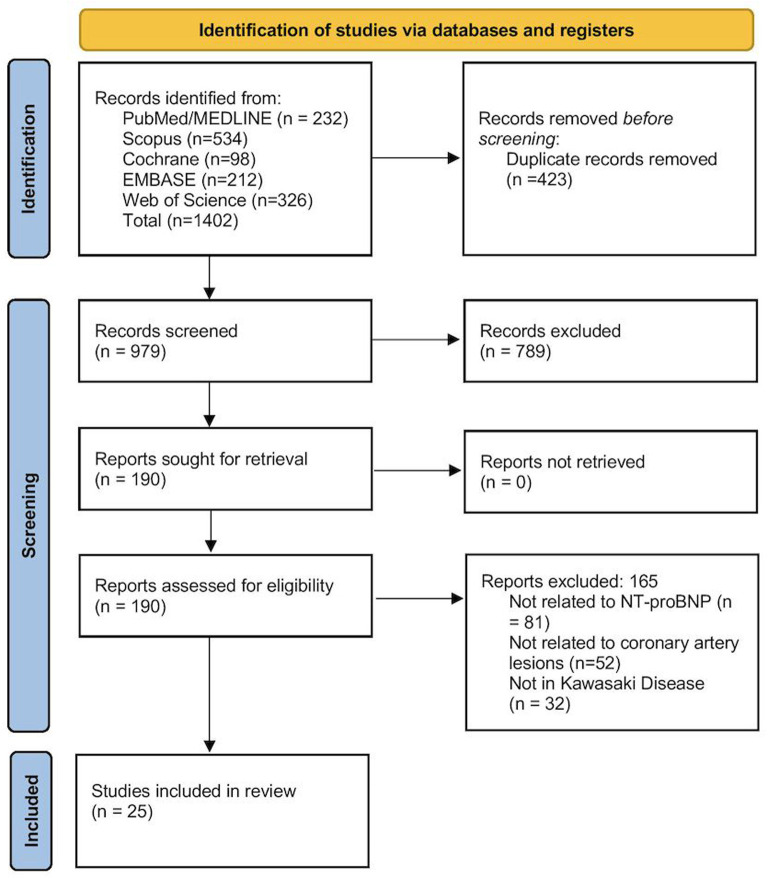
Preferred Reporting Items for Systematic Reviews and Meta-Analyses (PRISMA) flowchart showing the entire study selection process.

Titles and abstracts of identified papers were independently screened by the 2 reviewers. Full texts of potentially eligible articles were assessed using the predefined criteria (Figure 1). Differences were resolved by discussion.

### Data Extraction

Data that were independently retrieved by the 2 authors using a standardized form contained the following: author, year, state, design, cohort size, and diagnostic criteria for KD and CAL; participant characteristics such as age, sex, and baseline clinical features; and outcome measures of NT-proBNP levels in the form of either mean ± SD or median and range/IQR and diagnostic parameters for CAL detection.

### Quality Assessment

Study quality was assessed by Newcastle-Ottawa Scale (NOS) for observational studies.^19^ Newcastle-Ottawa Scale assesses each study for selection, comparability, and outcome/exposure. Studies scoring ≥6 stars are of high quality. Diagnostic studies were assessed by the Quality Assessment of Diagnostic Accuracy Studies (QUADAS-2) tool.^20^ Differences were resolved by discussion.

### Statistical Analysis

Data were pooled using standardized mean differences (SMD) with 95% confidence intervals (CIs) using the mean and SD of values reported in individual studies. In studies where NT-proBNP levels were reported as median (range or interquartile range [IQR]), the data were transformed into mean and SD values to ensure consistency. Due to anticipated heterogeneity, a random-effects model was selected. Heterogeneity was quantified using the *I*² statistic and the Cochran’s Q test:^21^ *I*² > 50% indicates significant heterogeneity.

A bivariate random-effects model was used for diagnostic accuracy, pooled sensitivity, specificity, positive likelihood ratio (LR), negative LR, and diagnostic odds ratio (DOR). Summary receiver-operating characteristic curves were constructed, and the AUROC was reported to evaluate overall diagnostic performance. An LR scattergram was plotted to determine the clinical value of NT-proBNP for CAL. A bivariate box plot was used to determine the heterogeneity.

Publication bias was assessed using a funnel plot and Egger’s and Deek’s tests. *P *< .05 was indicative of publication bias. STATA version 14.2 (StataCorp.; TX, USA) was used for analyses.

## Results

## Search Results

A total of 1402 records were retrieved, and 423 duplicates were removed. Following the screening, 789 articles were eliminated, and 190 full texts were reviewed for eligibility. Ultimately, 25 articles were included in the final analysis (Figure 1).^22-46^

### Characteristics of the Included Studies

As shown in Table 1, the included studies had diverse designs, with a mix of retrospective and prospective studies and cohort sizes from 12 to 5151 participants. Most studies were done in China, Korea, and Japan. The sensitivity and specificity for NT-proBNP in diagnosing CAL in KD patients were within the 0.412 to 1.0 and 0.378 to 1.0 range, respectively. The NT-proBNP cutoff levels used also varied widely. The risk of bias assessments varied across studies, with most (11 studies) having a lower risk of bias.

### N-Terminal Pro-Brain Natriuretic Peptide Levels in Patients with and Without Coronary Artery Lesion

**Figure 2.**
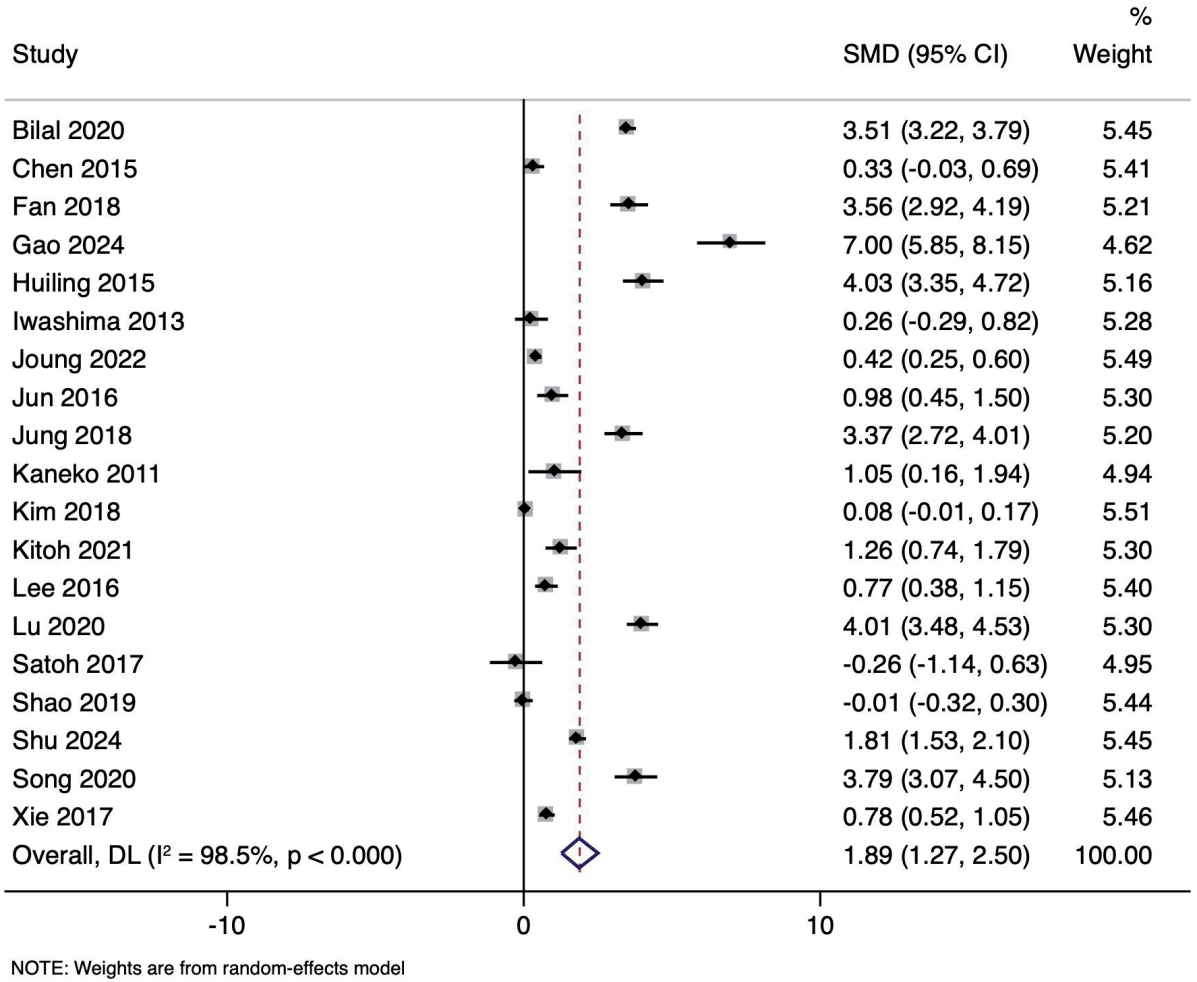
Forest plot showing the association between N-terminal pro-brain natriuretic peptide and coronary artery lesion in Kawasaki disease patients.

Nineteen studies involving 9017 participants compared the mean and SD of NT-proBNP levels in KD patients with/without CAL. The pooled SMD was 1.889 (95% CI: 1.274 to 2.504, *P* < .001), indicating considerably higher NT-proBNP levels in patients with CAL (Figure 2). Substantial heterogeneity was observed (*I*² = 98.5%, *P* < .001), with a tau² value of 1.7860. The funnel plot was asymmetrical (Supplementary Figure 1) and significant Egger’s test (*P* = .002) indicated the possibility of publication bias.

### Diagnostic Accuracy of N-Terminal Pro-Brain Natriuretic Peptide for Coronary Artery Lesion in Kawasaki Disease Patients

**Figure 3.**
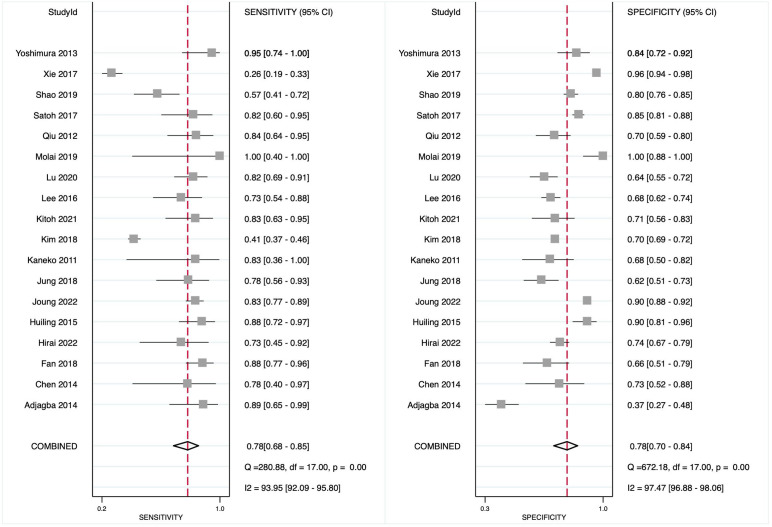
Forest plot showing the diagnostic accuracy of N-terminal pro-brain natriuretic peptide in detecting coronary artery lesion in Kawasaki disease patients.

**Figure 4.**
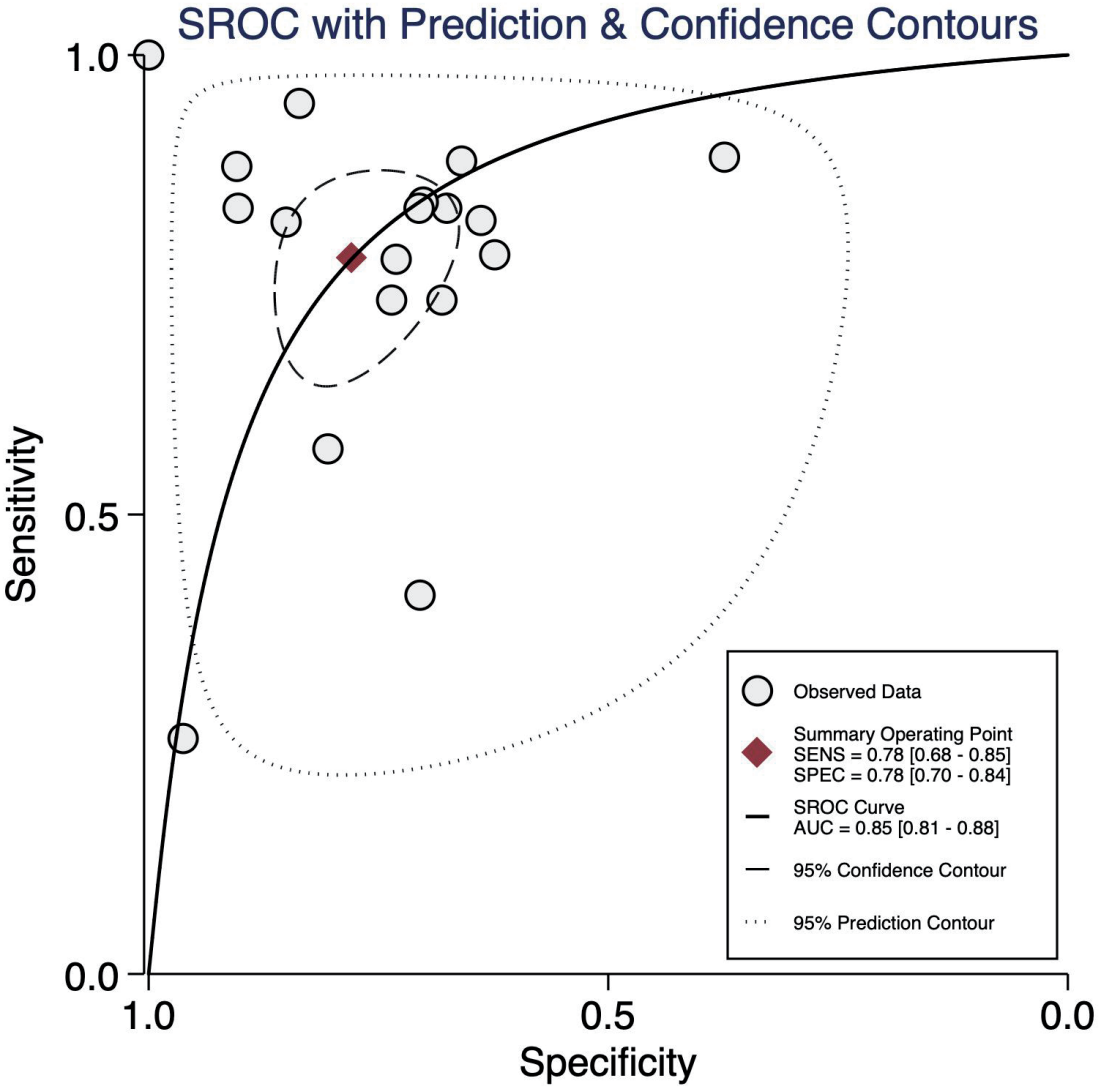
Summary Receiver Operator Characteristic curve for showing the accuracy of N-terminal pro-brain natriuretic peptide in detecting coronary artery lesion in Kawasaki disease patients.

Eighteen studies have reported on the diagnostic accuracy of NT-proBNP for CAL, with 1212 reference-positive and 7668 reference-negative units, with pooled sensitivity and specificity of 0.78 (95% CI: 0.68-0.85) and 0.78 (95% CI: 0.70-0.84), respectively (Figure 3). The diagnostic odds ratio (DOR) was 12 (95% CI: 7-21), with a positive LR of 3.5 (95% CI: 2.6-4.8) and a negative LR of 0.28 (95% CI: 0.20-0.41). The AUROC was 0.85 (95% CI: 0.81-0.88), indicating good diagnostic accuracy (Figure 4).

**Figure 5.**
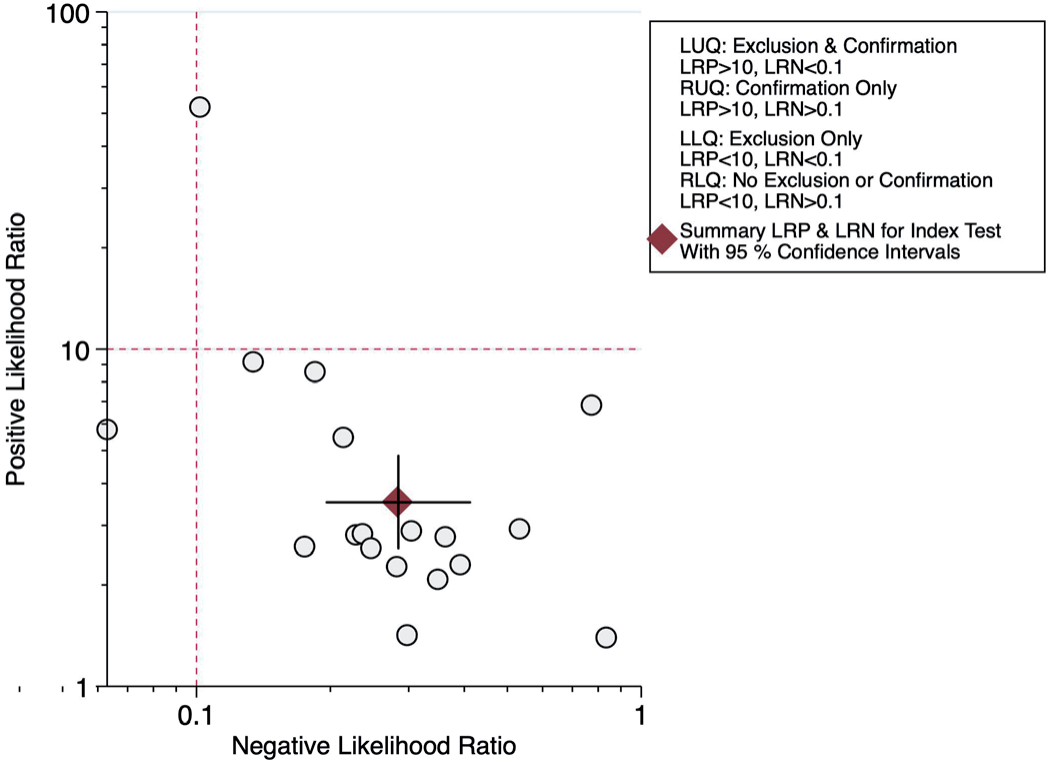
Likelihood ratio scattergram for N-terminal pro-brain natriuretic peptide in detecting coronary artery lesion in Kawasaki disease patients.

**Figure 6.**
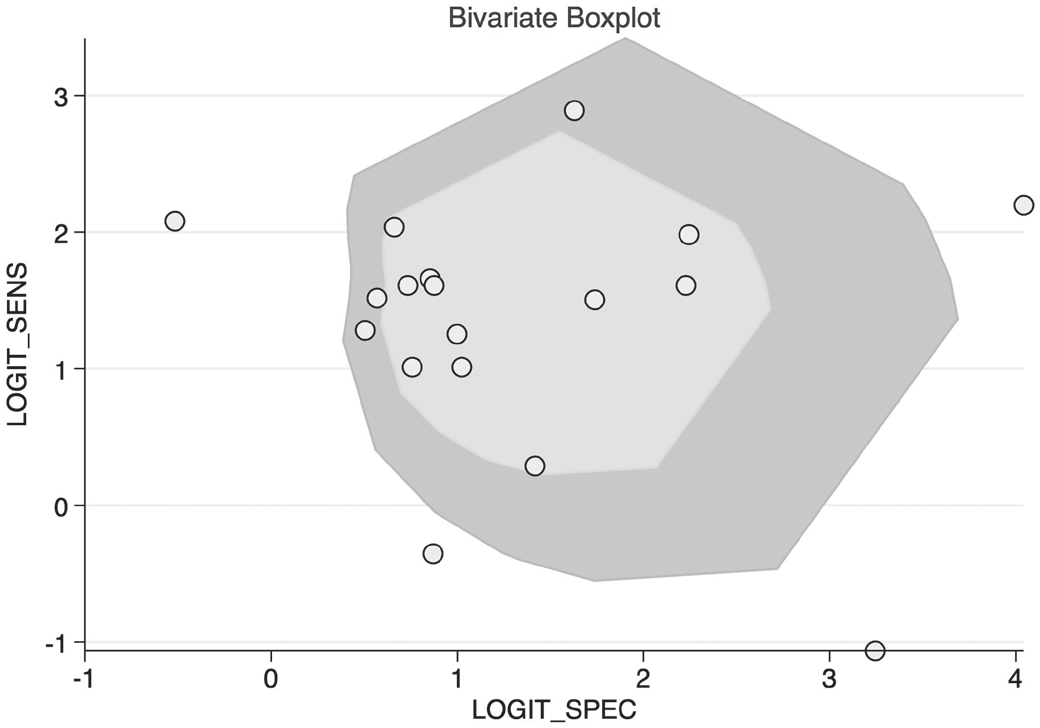
Bivariate boxplot for N-terminal pro-brain natriuretic peptide in detecting coronary artery lesion in Kawasaki disease patients.

An LR scattergram (Figure 5) demonstrated that the estimates were in the right lower quadrant, indicating that it cannot be used for either confirmation or exclusion. Substantial heterogeneity was observed (*I*² = 99%, *P* < .001), with a minor contribution from the threshold effect (proportion of heterogeneity due to threshold effect = 0.13) (Figure 6). The funnel plot (Supplementary Figure 2) was asymmetrical, and Deek’s test was significant (*P* = .01).

## Discussion

This review highlights significant findings regarding the association between NT-proBNP levels and CAL in KD among 25 studies. The pooled analysis of 19 studies demonstrated that KD complicated with CAL was associated with significantly higher NT-proBNP levels than KD without CAL, with a pooled SMD of 1.889. This finding suggests that NT-proBNP, a widely recognized biomarker for cardiac dysfunction, may be a valuable marker of coronary complications in KD patients. Additionally, the pooled diagnostic accuracy metrics from 18 studies revealed that NT-proBNP has moderate sensitivity and specificity (78% both) for identifying CAL, with an AUROC of 0.85, indicating good diagnostic performance. These results underscore the potential of NT-proBNP as a diagnostic marker for CAL in KD, though substantial heterogeneity and evidence of publication bias warrant cautious interpretation.

The elevated NT-proBNP levels observed in KD patients with CAL align with the underlying pathophysiology of KD, where systemic inflammation and endothelial dysfunction contribute to myocardial strain and coronary involvement.^47,48^ While similar findings have been reported in earlier studies, individual results varied widely. The previous meta-analysis by Zheng et al. focused on NT-proBNP in KD and reported significant associations but was limited by a smaller sample size (only 8 studies) and less rigorous methodological frameworks.^49^ The current study, with a broader scope and larger pooled sample, strengthens the evidence for this association while providing a more precise estimate of NT-proBNP’s diagnostic utility.

In terms of diagnostic accuracy, the observed AUROC of 0.85 corroborates earlier findings that NT-proBNP performs well as a diagnostic marker, though not sufficiently robust for standalone clinical use. A meta-analysis by Zheng et al. has reported sensitivities ranging from 65% to 85% and specificities from 60% to 90%, consistent with the pooled estimates of this study.^49^ However, as indicated by the LR scattergram, NT-proBNP cannot be solely relied upon for confirmation or exclusion, highlighting a limitation that previous studies often overlooked. This insight emphasizes the need for NT-proBNP to be integrated into broader diagnostic frameworks alongside other clinical and laboratory findings.

The higher NT-proBNP levels in KD patients with CAL can be attributed to the physiological response of myocardial strain and stress on the cardiovascular system caused by inflammatory processes in KD.^9^ N-terminal pro-brain natriuretic peptide, a peptide released from ventricular myocytes in response to increased wall stress, reflects cardiac dysfunction and is particularly elevated during episodes of systemic inflammation and vascular damage.^50^ In KD, the acute inflammatory phase results in endothelial dysfunction, coronary artery dilation, and myocardial involvement, all contributing to the increased release of NT-proBNP. Furthermore, the chronic sequelae of KD, including scarring and impaired coronary perfusion, may exacerbate these elevations in patients who develop CAL.^51^

The diagnostic utility of NT-proBNP is supported by its role as a biomarker for cardiac dysfunction, which is intricately linked to coronary artery abnormalities in KD.^6^ However, this study detected variability in diagnostic performance metrics across studies, which may stem from differences in NT-proBNP assay methods, thresholds, and patient populations, as well as the timing of biomarker measurement relative to the disease phase.^52^ These factors likely contribute to the substantial heterogeneity observed in the meta-analysis. Moreover, NT-proBNP levels may be potentially influenced by factors such as age, hydration status, and concurrent infections, which must also be considered when interpreting its diagnostic value.

The findings of this meta-analysis provide critical insights into the clinical utility of NT-proBNP in KD but also underscore the complexities of its application in diverse settings. While NT-proBNP levels are undeniably linked to CAL, their role in risk stratification and management must be interpreted within the context of individual patient characteristics and additional diagnostic tools.

This review has several notable strengths. First, it includes a comprehensive and rigorous analysis of 25 studies with a large pooled sample of nearly 10 000 participants. It provides, therefore, robust estimates of the relationship between NT-proBNP levels and CAL in KD. Second, the study employed standardized methodologies, including converting non-normally distributed data to mean (SD) by validated tools, ensuring consistency in data analysis. Third, the dual focus on both NT-proBNP levels and its diagnostic accuracy enhances the clinical applicability of the findings, offering valuable insights into the utility of this biomarker in diverse clinical scenarios.

The findings of this study have significant clinical value for managing KD patients. Elevated NT-proBNP levels in patients with CAL suggest that this biomarker may serve as an adjunctive tool for identifying children at higher risk of coronary complications. The good diagnostic accuracy (AUROC = 0.85) supports its use in early screening and risk stratification, particularly in settings with limited echocardiographic resources. However, the inability of NT-proBNP to independently confirm or exclude CAL, as indicated by the LR scattergram, highlights the need for its integration into a multimodal diagnostic approach. Combining NT-proBNP measurements with clinical assessments, imaging modalities, and other biomarkers may optimize identifying and managing high-risk patients. The study also reinforces the importance of timely biomarker assessment in KD. Elevated NT-proBNP levels during the acute phase of illness could help guide early interventions to mitigate long-term coronary damage. Additionally, NT-proBNP testing could facilitate the monitoring of therapeutic responses and the effectiveness of interventions to reduce coronary involvement in KD.

However, the study also has limitations. The substantial heterogeneity observed in the pooled analyses may limit the generalizability of the findings. This heterogeneity likely arises from differences in study populations, measurement methods, timing of NT-proBNP assessment, and thresholds for defining CAL. Additionally, the evidence of publication bias suggests a potential overestimation of the pooled effect sizes. Another limitation is the lack of subgroup analyses to account for factors such as age, severity of KD, and phase of illness, which may influence NT-proBNP levels. Finally, the reliance on observational studies, which are inherently prone to confounding, underscores the need for caution in interpreting causality.

Translating these findings into practice will require prospective validation of NT-proBNP cutoffs in well-defined pediatric cohorts and integration into existing diagnostic algorithms. In resource-limited settings where echocardiography is not readily available, an NT-proBNP–based triage step could prioritize high-risk children for urgent imaging. Cost-effectiveness studies should evaluate the economic and logistical feasibility of routine NT-proBNP screening in febrile children suspected of KD. Ultimately, guideline committees may consider incorporating NT-proBNP thresholds—stratified by age and assay platform—into KD management protocols to optimize early identification of CAL and improve long-term cardiovascular outcomes. Moreover, recent pharmacoeconomic work from the Kawarabi initiative demonstrates that region-specific cost-effectiveness analyses are essential for tailoring KD management to local resource constraints and healthcare structures. Finally, lessons drawn from the broader field of pediatric vasculitis—such as standardized outcome measures and long-term surveillance frameworks—offer valuable guidance for refining KD treatment protocols and ensuring comprehensive follow-up care.^53,54^

Future research should address the limitations identified in this meta-analysis to provide more conclusive evidence on the utility of NT-proBNP in KD. Large, multicenter prospective studies are needed to validate the findings and account for potential confounding factors such as age, phase of illness, and treatment regimens. Standardization of NT-proBNP measurement methods, including uniform thresholds and timing of assessment, will be critical to reducing inter-study variability and enhancing comparability across studies.

Further exploration of NT-proBNP’s role in combination with other biomarkers or diagnostic tools is warranted to develop a comprehensive risk stratification model for CAL in KD. Additionally, research into the pathophysiological mechanisms linking NT-proBNP and CAL may uncover new therapeutic targets and improve the understanding of KD-related coronary damage. Studies investigating the cost-effectiveness and feasibility of routine NT-proBNP testing in different healthcare settings would also help inform clinical guidelines.

This review provides compelling evidence that NT-proBNP levels are significantly elevated in KD patients with CAL compared to those without, highlighting its potential as a biomarker for coronary complications in KD. The diagnostic accuracy analysis demonstrates good performance, though NT-proBNP assessment should be part of a multimodal diagnostic framework rather than a standalone tool. Despite limitations such as heterogeneity and publication bias, the study underscores the relevance of NT-proBNP in KD management and calls for further research to refine its clinical application.

## Supplementary Materials

Supplementary Material

## Figures and Tables

**Table 1.** Study Level Characteristics and Risk of Bias Assessment of the Included Reports (N = 25)

Author and Year	Country	Study Design	Sample Size No CAL/CAL	Cutoff (pg/ml)	Mean Age	Sensitivity	Specificity	Diagnostic Criteria	Risk of Bias (NOS score)
Adjagba et al,^22^ 2014	Canada	Prospective	91/18	NR	NR	0.889	0.378	Established according to American Heart Association guideline.	Moderate (5)
Bilal et al,^23^ 2020	Pakistan	Observational	200/300	1.000	NR	NR	NR	Established according to American Heart Association guideline.	High (2)
Chen & Du^24^ 2014	China	Prospective	9/26	900	24.0/20.4	0.78	0.73	Established according to Chinese Medical Association guideline.	Moderate (4)
Chen et al,^25^ 2015	China	Prospective	83/47	NR	NR	NR	NR	Established according to Chinese Medical Association guideline.	Low (7)
Fan et al,^26 ^2018	China	Retrospective	52/47	NR	NR	87.5	66.7	Established according to Japan Kawasaki Disease Research Committee.	High (2)
Gao et al,^27 ^2024	China	Retrospective	53/32	NR	NR	NR	NR	Diagnostic criteria for children with KD selected in this study refer to criteria in Suggestions for Clinical Management of Coronary Arterions.	Low (8)
Hirai et al,^28 ^2022	Japan	Retrospective	227/15	1000, 900, 800, 700, 600, 500,400, 300	28.3 ± 22.7	0.733	0.833	Diagnosed based on the Diagnostic Guidelines for Kawasaki Disease (5th revised edition).	High (1)
Huiling et al,^29^ 2015	China	Prospective	73/33	950	NR	0.881	0.890	Established according to Japan Kawasaki Disease Research Committee.	Low (7)
Iwashima & Ishikawa,^30^ 2013	Japan	Retrospective	47/17	Less than 3 years old: <170.5 pg/mL, older than 3 years: <124.7 pg/mL.	1.6 ± 1.9	NR	NR	Fever exceeding 38°C accompanied by presence of at least 4 of following 5 findings: bilateral conjunctival injection, changes in lips and oral cavity, nonpurulent cervical lymphadenopathy, polymorphous exanthema, and changes in extremities.	High (3)
Joung et al,^31^ 2022	Korea	Retrospective	740/156	789	25 ± 22	0.835	0.903	Established according to American Heart Association guideline.	Low (8)
Jun et al,^32^ 2016	Korea	Retrospective	114/17	NR	48 ± 24 / 24 ± 12	0.78	0.84	Established according to American Heart Association guideline.	High (2)
Jung et al,^33^ 2018	Korea	Retrospective	86/23	515	NR	78.3	61.6	Established according to American Heart Association guideline.	Low (9)
Kaneko et al,^34^ 2011	Japan	Prospective	37/6	1.000	26.4/21.6	0.83	0.68	Diagnostic criteria established by Kawasaki disease research committee.	Moderate (4)
Kim et al,^35^ 2018	Korea	Retrospective	4627/524	709	NR	0.412	0.705	Established according to American Heart Association guideline 2004.	Low (8)
Kitoh et al,^36^ 2021	Japan	Prospective	51/24	453.2	21.2 ± 22.9	0.84	0.71	Fulfilled diagnostic criteria for KD as endorsed by Diagnostic Guidelines for Kawasaki Disease (5th revision).	Low (8)
Lee & Song,^37^ 2016	Korea	Retrospective	257/30	1.088	28.8/39.1	0.73	0.68	Established according to American Heart Association guideline 2004.	High (3)
Lu et al,^38^ 2020	China	Prospective	130/50	684	28 ± 22	0.82	0.65	Established according to American Heart Association guideline 2017.	Moderate (4)
Molaei et al,^39^ 2019	Iran	Cross-sectional	28/4	1.354	48 ± 12 / 24 ± 12	1.00	1.00	American Heart Association’s criteria for classic KD in children.	Low (7)
Qiu et al,^40^ 2012	China	Retrospective	77/25	827	21.5/13.3	0.803	0.84	Established according to American Heart Association guideline.	Moderate (5)
Satoh et al,^41^ 2017	Japan	Retrospective	388/22	1.555	34 ± 28 / 2 ± 1	0.80	0.85	Established by existence of high fever for at least 5 days and presence of at least 4 of following 5 criteria: (i) changes in extremities; (ii) changes in lips and oral cavity; (iii) polymorphous exanthema; (iv) bulbar conjunctival injection; and (v) cervical lymphadenopathy.	High (2)
Shao et al,^42^ 2019	China	Prospective	348/45	3755.0	NR	0.44	0.84	Standards recommended by the American Heart Association.	Low (7)
Shu et al,^43^ 2023	China	Retrospective	NR	NR	NR	NR	NR	Conformed to Kawasaki disease diagnostic criteria of 8th edition of ZHUFUTANG Practical Pediatrics.	Low (8)
Song et al,^44^ 2020	China	Comparative Study	72/23	NR	NR	NR	NR	Kawasaki Disease Diagnosis Guide-9.	Low (7)
Xie et al,^45^ 2017	China	Retrospective	407/153	1300.0	NR	0.73	0.76	Subjects had at least 5 of following 6 principal clinical signs: 1) fever persisting for 5/more days; 2) bilateral conjunctival congestion; 3) changes to lips and oral cavity; 4) polymorphous exanthema; 5) changes to peripheral extremities; and 6) acute nonpurulent cervical lymphadenopathy.	High (2)
Yoshimura et al,^46^ 2013	Japan	Prospective	61/19	1300	NR	0.94	0.83	Criteria for Diagnostic Guideline established by Kawasaki Disease Research Committee in Japan.	High (2)

KD, Kawasaki disease; NR, not reported.

## Data Availability

The data that support the findings of this study are available on request from the corresponding author.

## References

[1] Lo MS . A framework for understanding Kawasaki disease pathogenesis. Clin Immunol. 2020;214:108385. (10.1016/j.clim.2020.108385) 32173601

[2] Greco A De Virgilio A Rizzo MI , et al. Kawasaki disease: an evolving paradigm. Autoimmun Rev. 2015;14(8):703 709. (10.1016/j.autrev.2015.04.002) 25882057

[3] Agarwal S Agrawal DK . Kawasaki disease: etiopathogenesis and novel treatment strategies. Expert Rev Clin Immunol. 2017;13(3):247 258. (10.1080/1744666X.2017.1232165) 27590181 PMC5542821

[4] Lin M-T Sun L-C Wu E-T Wang J-K Lue H-C Wu M-H . Acute and late coronary outcomes in 1073 patients with Kawasaki disease with and without intravenous γ-immunoglobulin therapy. Arch Dis Child. 2015;100(6):542 547. (10.1136/archdischild-2014-306427) 25564534

[5] Dean M Kim MJ Dimauro S , et al. Cardiac and noncardiac biomarkers in patients undergoing anthracycline chemotherapy - a prospective analysis. Cardiooncology. 2023;9(1):23. (10.1186/s40959-023-00174-1) 37106424 PMC10133897

[6] Cao Z Jia Y Zhu B . BNP and NT-proBNP as diagnostic biomarkers for cardiac dysfunction in both clinical and forensic medicine. Int J Mol Sci. 2019;20(8):1820. (10.3390/ijms20081820) 31013779 PMC6515513

[7] Lee W Cheah CS Suhaini SA , et al. Clinical manifestations and laboratory findings of Kawasaki disease: beyond the classic diagnostic features. Medicina (Kaunas). 2022;58(6):734. (10.3390/medicina58060734) 35743997 PMC9227912

[8] Singh S Jindal AK Pilania RK . Diagnosis of Kawasaki disease. Int J Rheum Dis. 2018;21(1):36 44. (10.1111/1756-185X.13224) 29131549 PMC7159575

[9] Dionne A Dahdah N . A decade of NT-proBNP in acute Kawasaki disease, from physiological response to clinical relevance. Children (Basel). 2018;5(10):141. (10.3390/children5100141) 30322059 PMC6210997

[10] Parsanathan R Jain SK . Novel invasive and noninvasive cardiac-specific biomarkers in obesity and cardiovascular diseases. Metab Syndr Relat Disord. 2020;18(1):10 30. (10.1089/met.2019.0073) 31618136 PMC7041332

[11] Wang Y Li T . Advances in understanding Kawasaki disease-related immuno-inflammatory response and vascular endothelial dysfunction. Pediatr Investig. 2022;6(4):271 279. (10.1002/ped4.12341) PMC9789937 36582276

[12] Kato M Ayusawa M Watanabe H , et al. Cardiac function on 3-D speckle tracking imaging and cytokines in Kawasaki disease. Pediatr Int. 2018;60(4):342 348. (10.1111/ped.13521) 29350882

[13] Zhang C Chen S Bian Y , et al. Prediction of intravenous immunoglobulin retreatment in children with Kawasaki disease using models combining lymphocyte subset and cytokine profile in an East Asian cohort. Clin Transl Immunology. 2024;13(3):e1498. (10.1002/cti2.1498) 38481614 PMC10936231

[14] Cantinotti M Law Y Vittorini S , et al. The potential and limitations of plasma BNP measurement in the diagnosis, prognosis, and management of children with heart failure due to congenital cardiac disease: an update. Heart Fail Rev. 2014;19(6):727 742. (10.1007/s10741-014-9422-2) 24473828

[15] Uner A Doğan M Ay M Acar C . The evaluation of serum N-terminal prohormone brain-type natriuretic peptide, troponin-I, and high-sensitivity C-reactive protein levels in children with congenital heart disease. Hum Exp Toxicol. 2014;33(11):1158 1166. (10.1177/0960327113514097) 24501104

[16] Sarzani R Spannella F Giulietti F , et al. NT-proBNP and its correlation with in-hospital mortality in the very elderly without an admission diagnosis of heart failure. PLOS One. 2016;11(4):e0153759. (10.1371/journal.pone.0153759) 27077910 PMC4831737

[17] Méndez Hernández R Ramasco Rueda F . Biomarkers as prognostic predictors and therapeutic guide in critically ill patients: clinical evidence. J Pers Med. 2023;13(2):333. (10.3390/jpm13020333) 36836567 PMC9965041

[18] Chen C Chen Q Zhang T Ling Y . Coronary artery lesions in children with Kawasaki disease: status quo and nursing care. Front Cardiovasc Med. 2024;11:1272475. (10.3389/fcvm.2024.1272475) 38711795 PMC11070497

[19] Kansagara D O’Neil M Nugent S , et al. Quality assessment criteria for observational studies, based on the Newcastle-Ottawa scale [Internet]; 2017. Department of Veterans Affairs (US). Available at: https://www.ncbi.nlm.nih.gov/books/NBK476448/table/appc.t4/. Accessed 2024 Dec 31.

[20] Whiting PF Rutjes AWS Westwood ME , et al. QUADAS-2: a revised tool for the quality assessment of diagnostic accuracy studies. Ann Intern Med. 2011;155(8):529 536. (10.7326/0003-4819-155-8-201110180-00009) 22007046

[21] Cochrane handbook for systematic reviews of interventions [Internet]. Available at: https://training.cochrane.org/handbook. Accessed 2024 Dec 31.

[22] Adjagba PM Desjardins L Fournier A Spigelblatt L Montigny M Dahdah N . N-terminal pro-brain natriuretic peptide in acute Kawasaki disease correlates with coronary artery involvement. Cardiol Young. 2015;25(7):1311 1318. (10.1017/S1047951114002431) 25544036

[23] Bilal M Haseeb A Saeed A Sher Khan MA . The importance of serum N-terminal pro-brain natriuretic peptide and endogenous hydrogen sulfide for predicting coronary artery lesions in pediatric Kawasaki disease patients: findings from a Tertiary Care Hospital in Karachi, Pakistan. Cureus. 2020;12(7):e9016. (10.7759/cureus.9016) 32775096 PMC7405981

[24] Chen Y Du X . The value of N-terminal pro-brain natriuretic peptide in predicting coronary arterial lesions in Kawasaki disease. Guodong Medical [journal]. 2014:3075 3077.

[25] Chen T-T Shi K Liu Y-L Guo Y-H Li Y Wang X-M . Relationship between heart rate variability and coronary artery lesion in children with Kawasaki disease. Zhongguo Dang Dai Er Ke Za Zhi. 2015;17(6):607 612. 26108324

[26] Fan J Duan W Luo H Yang L Tao Y . Prediction of plasma NT-proBNP and PCT for early Kawasaki disease coronary artery lesions. J Pediatr Pharm. 2018;24:1 5.

[27] Gao W Chen Z Lu Y Bai X Chen M Lu Y . Analysis of ultrasound coronary parameters and blood red cell distribution width and N-terminal pro-brain natriuretic peptide concentrations following coronary lesions in children with Kawasaki disease. Br J Hosp Med (Lond). 2024;85:1 10. 10.12968/hmed.2024.0162 39212580

[28] Hirai S Nakamura T Misawa M . Predictive potential of age-group cut-off values of N-terminal pro-brain natriuretic peptide in Kawasaki disease. Pediatr Int. 2022;64(1):e15371. (10.1111/ped.15371) 36166642

[29] Huiling L Yaping L Xiufen H . Prediction of the risk of coronary arterial lesions in Kawasaki disease by N-terminal pro-brain natriuretic peptide. Zhonghua Er Ke Za Zhi. 2015;53(4):300 303. 26182507

[30] Iwashima S Ishikawa T . B-type natriuretic peptide and N-terminal pro-BNP in the acute phase of Kawasaki disease. World J Pediatr. 2013;9(3):239 244. (10.1007/s12519-013-0402-8) 23335186

[31] Joung J Oh JS Yoon JM Ko KO Yoo GH Cheon EJ . A decision tree model for predicting intravenous immunoglobulin resistance and coronary artery involvement in Kawasaki disease. BMC Pediatr. 2022;22(1):474. (10.1186/s12887-022-03533-6) 35931986 PMC9354345

[32] Jun H Ko KO Lim JW Yoon JM Lee GM Cheon EJ . Age-adjusted plasma N-terminal pro-brain natriuretic peptide level in Kawasaki disease. Korean J Pediatr. 2016;59(7):298 302. (10.3345/kjp.2016.59.7.298) 27588030 PMC5007425

[33] Jung JY Ham EM Kwon H , et al. N-terminal pro-brain natriuretic peptide and prediction of coronary artery dilatation in hyperacute phase of Kawasaki disease. Am J Emerg Med. 2019;37(3):468 471. (10.1016/j.ajem.2018.06.021) 29903669

[34] Kaneko K Yoshimura K Ohashi A Kimata T Shimo T Tsuji S . Prediction of the risk of coronary arterial lesions in Kawasaki disease by brain natriuretic peptide. Pediatr Cardiol. 2011;32(8):1106 1109. (10.1007/s00246-011-9986-8) 21487793

[35] Kim MK Song MS Kim GB . Factors predicting resistance to intravenous immunoglobulin treatment and coronary artery lesion in patients with Kawasaki disease: analysis of the Korean nationwide multicenter survey from 2012 to 2014. Korean Circ J. 2018;48(1):71 79. (10.4070/kcj.2017.0136) 29171205 PMC5764872

[36] Kitoh T Ohara T Muto T , et al. Increased PENTRAXIN 3 levels correlate with IVIG responsiveness and coronary artery aneurysm formation in Kawasaki disease. Front Immunol. 2021;12:624802. (10.3389/fimmu.2021.624802) 33912155 PMC8072470

[37] Lee HY Song MS . Predictive factors of resistance to intravenous immunoglobulin and coronary artery lesions in Kawasaki disease. Korean J Pediatr. 2016;59(12):477 482. (10.3345/kjp.2016.59.12.477) 28194213 PMC5300912

[38] Lu Y Guo Y Si F , et al. Predictive value of heart rate deceleration capacity on coronary artery lesion in acute phase of Kawasaki disease. Sci Rep. 2020;10(1):10211. (10.1038/s41598-020-67121-3) 32576944 PMC7311450

[39] Molaei A Sadeghi-Shabestari M Khomahani A Ghaffari S Sadat-Ebrahimi S-R . Cardiac biomarkers for early detection of cardiac involvement in children with Kawasaki disease: A cross-sectional study. J Pediatr Perspect. 2019;7:10573 10582.

[40] Qiu H Ruan M Chen Q Zhang Y Wu R Xiang R . Prediction of the Risk of Coronary arterial Lesions in Kawasaki disease by N - terminal Brain Natriuretie Peptide. J Med Res. 2012;4:166 168.

[41] Satoh K Wakejima Y Gau M , et al. Risk of coronary artery lesions in young infants with Kawasaki disease: need for a new diagnostic method. Int J Rheum Dis. 2018;21(3):746 754. (10.1111/1756-185X.13223) 29105337

[42] Shao S Luo C Zhou K , et al. The role of age-specific N-terminal pro-brain natriuretic peptide cutoff values in predicting intravenous immunoglobulin resistance in Kawasaki disease: a prospective cohort study. Pediatr Rheumatol Online J. 2019;17(1):65. (10.1186/s12969-019-0368-8) 31533770 PMC6751871

[43] Shu Z Deng F Yang S . Early clinical evaluation of coronary artery lesions in Kawasaki disease. Clin Pediatr (Phila). 2024;63(9):1287 1291. (10.1177/00099228231219501) 38135926

[44] Song H-B Zhang YD Dong Q-W , et al. Significance of serum NT-proBNP and endogenous H₂S for predicting coronary artery lesions in pediatric Kawasaki disease. J Coll Physicians Surg Pak. 2020;30(1):37 40. (10.29271/jcpsp.2020.01.37) 31931930

[45] Xie T Wang Y Fu S , et al. Predictors for intravenous immunoglobulin resistance and coronary artery lesions in Kawasaki disease. Pediatr Rheumatol Online J. 2017;15(1):17. (10.1186/s12969-017-0149-1) 28320400 PMC5359815

[46] Yoshimura K Kimata T Mine K Uchiyama T Tsuji S Kaneko K . N-terminal pro-brain natriuretic peptide and risk of coronary artery lesions and resistance to intravenous immunoglobulin in Kawasaki disease. J Pediatr. 2013;162(6):1205 1209. (10.1016/j.jpeds.2012.11.026) 23290510

[47] Pilania RK Jindal AK Bhattarai D Naganur SH Singh S . Cardiovascular involvement in Kawasaki disease is much more than mere coronary arteritis. Front Pediatr. 2020;8:526969. (10.3389/fped.2020.526969) 33072669 PMC7542237

[48] Selamet Tierney ES Gal D Gauvreau K , et al. Vascular health in Kawasaki disease. J Am Coll Cardiol. 2013;62(12):1114 1121. (10.1016/j.jacc.2013.04.090) 23835006

[49] Zheng X Zhang Y Liu L , et al. N-terminal pro-brain natriuretic peptide as a biomarker for predicting coronary artery lesion of Kawasaki disease. Sci Rep. 2020;10(1):5130. (10.1038/s41598-020-62043-6) 32198398 PMC7083930

[50] Panagopoulou V Deftereos S Kossyvakis C , et al. NTproBNP: an important biomarker in cardiac diseases. Curr Top Med Chem. 2013;13(2):82 94. (10.2174/1568026611313020002) 23470072

[51] Lo M-H Lin Y-J Kuo H-C , et al. Assessment of vascular and endothelial function in Kawasaki disease. Biomed J. 2023;46(2):100525. (10.1016/j.bj.2022.03.010) 35358713 PMC10267959

[52] Schmitt W Rühs H Burghaus R , et al. NT-proBNP qualifies as a surrogate for clinical end points in heart failure. Clin Pharmacol Ther. 2021;110(2):498 507. (10.1002/cpt.2222) 33630302 PMC8360001

[53] Nait-Ladjemil D Harahsheh AS Choueiter N , et al. Pharmacoeconomic analysis and considerations for the management of Kawasaki disease in the Arab countries-A multinational, multi-institutional project of the Kawasaki disease Arab initiative (Kawarabi) (A project methodology paper). Turk Arch Pediatr. 2025;60(2):172 181. (10.5152/TurkArchPediatr.2025.24248) 40094277 PMC11963343

[54] Barut K Sahin S Kasapcopur O . Pediatric vasculitis. Curr Opin Rheumatol. 2016;28(1):29 38. (10.1097/BOR.0000000000000236) 26555448

