# Supplementary material for: Association Between Levels of N-Terminal Pro-brain Natriuretic Peptide and Coronary Artery Lesion in Patients with Kawasaki Disease: A Systematic Review and Meta-analysis
Source: Arch Rheumatol. 2025 Jun 23;40(2):256–66. doi: 10.5152/ArchRheumatol.2025.11128 (PMC12260449; doi:10.5152/ArchRheumatol.2025.11128)
Supplement: Supplementary Material [file supplementary_material.pdf]

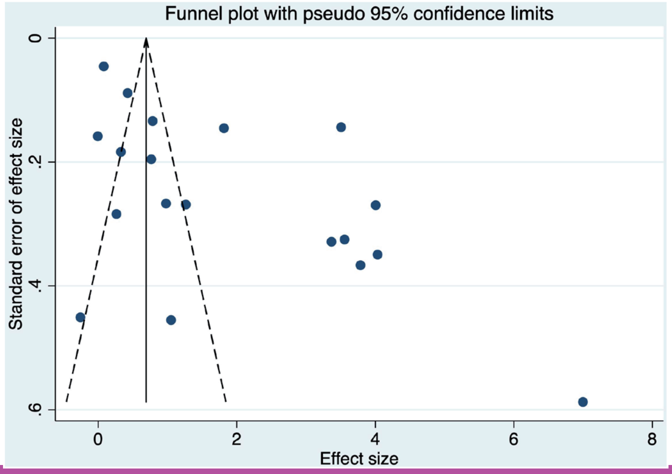

Supplementary Figure 1. Funnel plot for association between NT-proBNP and CAL in Kawasaki disease patients.

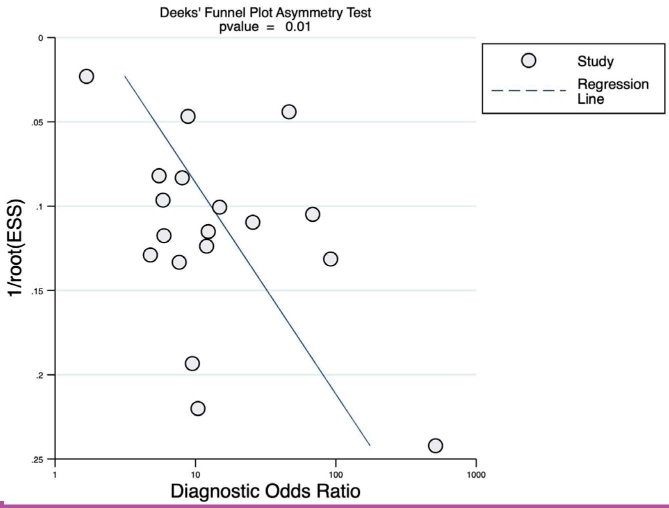

Supplementary Figure 2. Funnel plot for diagnostic accuracy of NT-proBNP in detecting CAL in Kawasaki disease patients.
